# Application of Density Functional Theory to Molecular Engineering of Pharmaceutical Formulations

**DOI:** 10.3390/ijms26073262

**Published:** 2025-04-01

**Authors:** Haoyue Guan, Huimin Sun, Xia Zhao

**Affiliations:** National Institute for Food and Drug Control, Beijing 100050, China; guanhaoyue@nifdc.org.cn

**Keywords:** density functional theory, pharmaceutical formulations, molecular interactions, drug release

## Abstract

This review systematically examines the pivotal applications of the Density Functional Theory (DFT) in drug formulation design, emphasizing its capability to elucidate molecular interaction mechanisms through quantum mechanical calculations. By solving the Kohn–Sham equations with precision up to 0.1 kcal/mol, DFT enables accurate electronic structure reconstruction, providing theoretical guidance for optimizing drug–excipient composite systems. In solid dosage forms, DFT clarifies the electronic driving forces governing active pharmaceutical ingredient (API)–excipient co-crystallization, predicting reactive sites and guiding stability-oriented co-crystal design. For nanodelivery systems, DFT optimizes carrier surface charge distribution through van der Waals interactions and π-π stacking energy calculations, thereby enhancing targeting efficiency. Furthermore, DFT combined with solvation models (e.g., COSMO) quantitatively evaluates polar environmental effects on drug release kinetics, delivering critical thermodynamic parameters (e.g., ΔG) for controlled-release formulation development. Notably, DFT-driven co-crystal thermodynamic analysis and pH-responsive release mechanism modeling substantially reduce experimental validation cycles. While DFT faces challenges in dynamic simulations of complex solvent environments, its integration with molecular mechanics and multiscale frameworks has achieved computational breakthroughs. This work offers interdisciplinary methodology support for accelerating data-driven formulation design.

## 1. Introduction

In modern pharmaceutical formulation development, precision design at the molecular level is increasingly replacing traditional empirical trial-and-error approaches. This paradigm shift is driven by advancements in computational pharmaceutics. The Density Functional Theory (DFT), a fundamental aspect of quantum chemistry, offers transformative theoretical insights by elucidating the electronic nature of molecular interactions. This enables a systematic understanding of complex behaviors in drug–excipient composite systems. Significantly, more than 60% of formulation failures in the development of Biopharmaceutics Classification System (BCS) II/IV drugs are attributed to unforeseen molecular interactions between active pharmaceutical ingredients (APIs) and excipients [1]. This critical challenge underscores the limitations of traditional characterization techniques, where the absence of molecular descriptors forces reliance on extensive experimental iterations for stability prediction and dissolution modulation, severely impeding the advancement of novel delivery systems.

DFT effectively addresses these gaps through its remarkable ability to resolve electronic structures. By solving the Kohn–Sham equations with quantum mechanical precision, achieving an accuracy of approximately 0.1 kcal/mol [2], DFT reconstructs molecular orbital interactions and demonstrates multifaceted applications: (1) in solid dosage forms, it deciphers electronic driving forces governing API–excipient co-crystallization, leveraging Fukui functions to predict reactive sites and guide stability optimization; (2) for nanodelivery systems, it enables precise calculation of van der Waals interactions and π-π stacking energy levels to engineer carriers with tailored surface charge distributions, and (3) in biomembrane transport simulations, the Fragment Molecular Orbital (FMO) theory quantifies energy barriers for drug permeation across phospholipid bilayers, thereby establishing quantitative structure–property relationships (QSPR) to enhance bioavailability.

Despite its widespread application, the implementation of DFT encounters several challenges. Current approximations in solvation modeling often fail to accurately represent the effects of polar environments. Additionally, the methodological limitations in simulating dynamic non-equilibrium processes pose significant constraints. Furthermore, achieving an optimal balance between computational cost and accuracy in multicomponent systems necessitates further refinement. Nevertheless, the advent of machine learning-augmented high-throughput DFT frameworks holds promise, as they represent transformative tools for advancing the digitalization of molecular engineering in formulation science.

**Figure 1:**
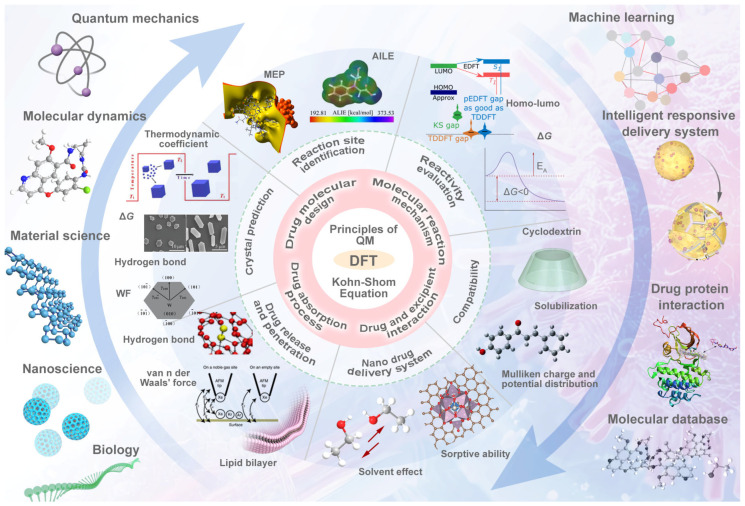
TOC (Reprinted with permission from Ref. [3], Copyright © 2021 by Deghady, A.M.; Hussein, R.K.; Alhamzani, A.G.; Mera, A.; Ref. [4]. Copyright © 2016 by Wiley-VCH Verlag GmbH & Co. KGaA, Weinheim. Ref. [5]. Copyright © 2016 by Kawai, S.; Foster, A.S.; Björkman, T.; Nowakowska, S.; Björk, J.; Canova, F.F.; Gade, L.H.; Jung, T.A.; Meyer, E; Ref. [6]. Copyright © 2015 by WILEY-VCH Verlag GmbH & Co. KGaA, Weinheim; Ref. [7]. Copyright © 2023 by Elsevier Ltd; Ref. [8]. Copyright © 2022 by American Chemical Society; Ref. [9]. Copyright © 2021 by Ran Friedman; Ref. [10]. Copyright © 2021 by American Chemical Society; Ref. [11]. Copyright © 2021 by American Chemical Society. Ref. [12] Copyright © 2023 Elsevier Inc.)

This review critically examines DFT’s evolving role in pharmaceutical formulation development, analyzing its capacity to elucidate intricate drug–excipient interactions while integrating experimental data to holistically assess formulation performance (see Figure 1). Through systematic analysis of recent advancements, we delineate how DFT is reconfiguring the drug development pipeline—from molecular design to formulation optimization—and project its future application.

## 2. Theoretical Foundations and Recent Advances in DFT

### 2.1. Fundamental Principles of DFT

DFT is a computational method based on the principles of quantum mechanics that describes the properties of multi-electron systems through electron density. DFT fully embodies the basic principles of quantum mechanical calculations by employing the Hohenberg–Kohn theorem, Kohn–Sham equations, the self-consistent field (SCF) method, as well as the selection of functionals and basis sets. The Hohenberg–Kohn theorem is the cornerstone of DFT, stating that the ground-state properties of a system are uniquely determined by its electron density. This conclusion, rooted in the principles of quantum mechanics, provides a solid theoretical foundation for DFT. By simplifying the complex multi-electron problem into a functional of electron density, the theorem avoids the complexity of directly solving the Schrödinger equation [13]. The Kohn–Sham equations reduce the multi-electron problem to a single-electron approximation by using a framework of non-interacting particles to reconstruct the electron density distribution of the real system. The kinetic energy term, electron-nuclear attraction term, classical Coulomb repulsion term, and exchange–correlation term in the Kohn–Sham equations all reflect quantum mechanical calculations. In particular, the exchange–correlation term, which encompasses the exchange and correlation effects of quantum mechanics, is the key feature that distinguishes DFT from classical theories [14].

DFT typically employs the self-consistent field (SCF) method, which iteratively optimizes the Kohn–Sham orbitals until convergence is achieved, yielding ground-state electronic structure parameters. These parameters include molecular orbital energies, geometric configurations (bond lengths and angles), vibrational frequencies, and dipole moments, all of which are calculated based on the principles of quantum mechanics. These parameters support the analysis of structure-activity relationships in drug molecules.

The accuracy of DFT is critically dependent on the selection of functionals and basis sets. Factors such as the size of the basis set, limitations of the dataset, the quality of model construction, and numerical convergence play significant roles in determining computational precision [15]. For instance, the Local Density Approximation (LDA) excels in metallic systems but inadequately describes weak interactions like hydrogen bonding and van der Waals forces. The Generalized Gradient Approximation (GGA) addresses this limitation by incorporating density gradient corrections, making it a preferred choice for biomolecular systems [16]. Furthermore, integrating solvation effects (e.g., via the COSMO model) enhances the reliability of thermodynamic parameter calculations by simulating solvent polarity influences through continuous dielectric medium models [15].

### 2.2. The Role of DFT in Multidisciplinary Applications

DFT calculations utilize a diverse array of exchange–correlation functionals, which are systematically classified into various tiers according to their methodological construction, spanning from nonempirical to empirical approaches. This classification encompasses the more rudimentary local density approximations (LDA), the more accurate generalized gradient approximations (GGA), meta-GGA, and hybrid functionals, and extends to more advanced formulations.

#### 2.2.1. Functional Diversity and Multi-Scenario Adaptability

The selection of functionals depends on their respective strengths and limitations, which vary according to the specific research context. For instance:

LDA is suitable for calculating crystal structures and simple metallic systems [17,18]; GGA is widely applied to molecular property calculations, hydrogen bonding systems, and surface/interface studies [19,20,21,22]; Time-dependent DFT (T-DFT) is particularly effective for investigating photocatalytic reactions, including excited-state reactivity and energy transfer processes [23,24,25]; Long-range corrected DFT (LC-DFT) is ideal for studying solvent effects, hydrogen bonding, van der Waals interactions, and the structure–function relationships of biomacromolecules [26,27,28]; Meta-GGA provides accurate descriptions of atomization energies, chemical bond properties, and complex molecular systems [29,30]; and Hybrid density functionals, such as B3LYP and PBE0, are widely employed for studying reaction mechanisms and molecular spectroscopy [31,32].

#### 2.2.2. Integration of Multiscale Computational Paradigms

The integration of DFT with machine learning (ML) and molecular mechanics (MM) has emerged as a significant trend in computational chemistry. Noteworthy examples include the following:

The ONIOM multiscale framework, where Zeyin Yan et al. employed DFT for high-precision calculations of drug molecule core regions while using MM force fields to model protein environments. This approach, combined with machine learning potentials (MLPs), resulted in a substantial enhancement in computational efficiency [33].

In the realm of deep learning-driven reaction prediction, David F. Nippa’s team utilized DFT-derived atomic charges to develop datasets for training geometric deep learning models (GNNs). These models successfully predicted reaction yields and regioselectivity of drug molecules. The GNN model was successfully applied to F. Hoffmann-La Roche Ltd.’s drug discovery program through the LSF platform. The LSF platform predicts reaction yields with an average absolute error of 4–5%. The platform achieved an activity classification accuracy of 92% for known substrates and 67% for unknown substrates in new reactions. Additionally, the regioselectivity prediction accuracy for major products was 67%, and it was successfully validated on 23 different commercial drug molecules [34].

#### 2.2.3. Innovations in Functionals and Applications

Recent advancements in DFT have been characterized by two major trends: the enhancement of high-precision functionals and the integration of multiscale computational frameworks. In terms of functional innovation, Double Hybrid Functionals, such as DSD-PBEP 86, incorporate second-order perturbation theory corrections, thereby substantially improving the accuracy of calculations related to excited-state energies and reaction barrier calculations [35,36,37]. Additionally, deep learning models have been used to approximate kinetic energy density functionals, as exemplified by M-OFDFT [38]. By using the Hartree–Fock density instead of the Kohn–Sham density transformation method, the calculation accuracy of water solvent environmental binding energy is significantly improved, and the derived MB-SCAN (DC) potential energy function can accurately describe the water phase transition [39]. The MB-SCAN (DC) potential energy function, derived from DC-SCAN MBE data, offers a marked improvement in accurately characterizing the properties of water across gaseous and liquid phases compared to previous DFT-based models, such as SCAN-AIMD and SCAN-NNP.

## 3. DFT in Drug Molecule Design and Reaction Mechanism Studies

DFT has emerged as an essential instrument in the design of drug molecules and the investigation of reaction mechanisms, owing to its precise capability in resolving electronic structures. DFT exhibits distinct advantages in the realms of drug activity prediction, mechanistic studies, and crystalline stability analysis. These advantages are explored through three dimensions: molecular reaction identification, reaction activity evaluation, and the regulation of solid-state properties.

### 3.1. Reaction Site Identification: Analysis Based on Electronic Distribution Features

Molecular Electrostatic Potential (MEP) maps and Average Local Ionization Energy (ALIE) are critical parameters for predicting drug–target binding sites. MEP maps depict the distribution of molecular surface charges by calculating electrostatic potentials (ESP), thereby identifying regions that are electron-rich (nucleophilic) and electron-deficient (electrophilic). ALIE quantifies the energies required for electron removal by weighting orbital energies, thus identifying the most vulnerable sites to electrophilic attacks. The combined analysis of these parameters provides insights into the spatial distribution of nucleophilic and electrophilic hotspots within drug molecules.

In MEP maps, distinct positive (blue) and negative (red) electrostatic potential regions correspond to the donor and receptor characteristics of the molecule, respectively. Regions depicted in red, indicating negative electrostatic potential (ESP), are associated with high electron density and are typically linked to electronegative atoms, such as oxygen, which exhibit nucleophilic properties. Conversely, blue regions, indicative of positive ESP, represent electron-deficient areas, such as hydrogen atoms bonded to oxygen, which are susceptible to electrophilic interactions. Neutral potentials are represented by green areas. In their study, Erdogan et al. [40] employed MEP maps to analyze charge distributions in eight natural products, revealing oxygen atoms as primary electron-rich sites critical for hydrogen bonding with the His41 residue of SARS-CoV-2’s main protease. This electronic complementarity is fundamental to the observed differences in binding affinity.

**Figure 2:**
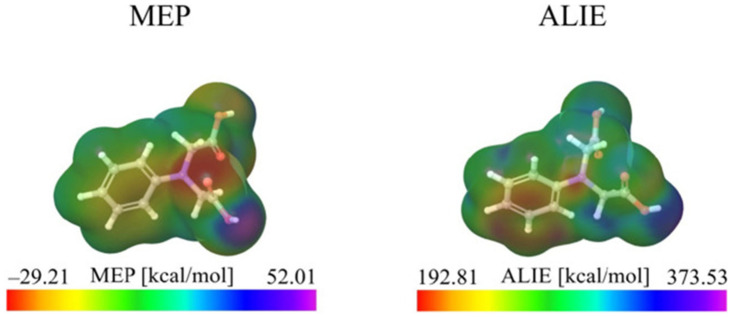
MEP and ALIE surfaces of PIDAA. Reproduced from ref. [41]. This is an open-access publication.

Similarly, ALIE maps utilize color coding to represent electron binding strength: regions shaded in red (indicating low ALIE values) correspond to weakly bound electrons that are susceptible to electrophilic attacks, such as those found in heteroatoms or unsaturated bonds. Conversely, regions shaded in blue (indicating high ALIE values) denote stable electron configurations, such as those present in alkyl chains or aromatic carbons. The green areas represent regions with moderate ALIE values. Christin et al. [41] integrated MEP and ALIE analyses (as shown in Figure 2) to predict reactive sites, identifying oxygen atoms as electrophilic targets and aromatic carbons/nitrogens as zones with weak electron binding. Mary et al. [42] applied dual MEP/ALIE analysis with the 3LYP/6-311G(d,p) basis set to identify sulfur as the active site in 1-(2-fluorophenyl)-3-[3-(trifluoromethyl)phenyl]thiourea (FPTT). Additionally, FPTT exhibited 92% solubility parameter compatibility with polyvinylpyrrolidone (PVP), facilitating the rational selection of excipients. Deka et al. [43] leveraged MEP in DFT studies to probe chitosan–nucleotide interactions. During the formation of chitosan–nucleotide adducts, hydrogen bonds were observed between the amino hydrogen atoms (-NH3) of chitosan and the oxygen/nitrogen atoms of nucleotides, illustrating the effectiveness of MEP in elucidating biopolymer interactions.

### 3.2. Reactivity Assessment: HOMO-LUMO Energy Gap and Global Reactivity Parameters

The highest occupied molecular orbital (HOMO) denotes the molecular orbital of the highest energy that is fully occupied by electrons, whereas the lowest unoccupied molecular orbital (LUMO) represents the molecular orbital of the lowest energy that remains unoccupied. The spatial distribution of the HOMO provides insights into the regions of a molecule most likely to engage in bond formation, while the LUMO distribution identifies areas susceptible to electron acceptance. Eg, the energy difference between HOMO and LUMO, is a critical parameter for assessing molecular reactivity. A smaller Eg (typically < 4 eV) correlates with higher chemical reactivity. By integrating Eg with global parameters such as chemical hardness (η) and electronegativity (χ), a comprehensive multidimensional framework for assessing reactivity can be developed.

In the field of drug design, the B3LYP functional is commonly utilized for high-throughput screening of molecular reactivity, particularly through the rapid evaluation of the HOMO–LUMO energy gap across various molecules. For example, Ayub et al. [43] investigated the HOMO–LUMO gaps of five pharmaceutical compounds to assess their potential efficacy against breast cancer. The findings indicated that lapatinib exhibited the smallest HOMO–LUMO gap (3.822 eV), suggesting higher reactivity, whereas anastrozole displayed the largest gap (6.113 eV), implying lower reactivity. Similarly, Gheidari et al. [44] conducted a screening of nine indeno [1,2-b] pyrrole-4(1H)-carboxamide derivatives to identify the most effective compound for inhibiting SARS-CoV-2’s Mpro. Among these, compound 5d was identified as having the smallest energy gap (3.9638 eV), the lowest hardness (1.9819), and the highest electronegativity (5.4594). In another study, Babu et al. [45] screened pyridine carboxamide derivatives and determined that compound 3 h possessed the largest energy gap and exhibited the strongest biological activity. Furthermore, Catalina et al. [12] demonstrated that substituting isoleucine with valine in a peptide sequence resulted in a reduced HOMO–LUMO energy gap, indicating enhanced molecular reactivity and facilitating electron excitation following valine substitution.

Despite its previously mentioned applications, recent research has identified systematic inaccuracies in B3LYP’s predictions of the absolute energies of HOMO and LUMO. These inaccuracies primarily arise from the fact that, despite incorporating a portion of exact exchange (approximately 20% Hartree–Fock exchange) to alleviate the self-interaction error (SIE), the fixed proportion is insufficient to fully compensate for long-range Coulomb repulsion. This results in an overly rapid decay of the asymptotic potential and a systematic underestimation of excitation energies.

In recent years, the advancement of range-separated hybrid functionals has introduced novel methodologies to address these limitations. Enhanced techniques, exemplified by optimally tuned range-separated hybrid (RSH) methods, combine exact long-range exchange and determine the range separation parameter ab initio based on the ionization potential theorem. It has been proven to accurately reproduce fundamental and optical gaps in finite-sized systems, including challenging charge-transfer scenarios [46,47]. Moreover, time-dependent density functional theory (TDDFT) calculations employing range-separated functionals, such as those adopted by Wong and Hsieh ([48]) in their study of oligoacenes, have provided significant improvements in predicting optoelectronic and excitonic properties. The long-range corrected functional ωB97XD, which includes dispersion corrections (D3 corrections), specifically addresses the shortcomings of traditional functionals concerning long-range electron correlation and dispersion forces. The study demonstrates that the use of ωB97XD results in more precise HOMO/LUMO energy calculations than B3YLP for the drug boceprevir [49]. These advancements highlight the importance of incorporating long-range exchange and system-specific tuning in DFT calculations, offering a more reliable alternative to traditional functionals (e.g., B3LYP) for accurately predicting electronic and optical properties in molecular systems.

### 3.3. Role Mechanism Analysis: From E-Localisation to Dynamic Interactions

The Electron Localization Function (ELF), Localized Orbital Locator (LOL), and Natural Bond Orbital (NBO) analyses provide insights into the stabilization mechanisms of drug–target complexes. ELF, which quantifies electron localization in space on a scale from 0 to 1, is particularly effective in characterizing lone pairs and bonding electron distributions. It is extensively applied in the analysis of bonds, electron pair localization, and the study of reaction mechanisms. Similarly, LOL, also ranging from 0 to 1, concentrates on electron localization within molecular orbitals and is widely used to study molecular interactions, such as hydrogen bonding and van der Waals forces. NBO analysis investigates intramolecular electron transfers and conjugation effects, offering detailed insights into electronic distributions and bonding properties. Through NBO, researchers can identify electron transfer pathways and conjugative interactions, helping to elucidate molecular stability and reactivity.

DFT-derived ELF and LOL reveal insights into the electronic characteristics of chemical bonds. In organic amine molecules, for example, ELF indicates highly localized electrons between aromatic carbon atoms, whereas oxygen atoms in carboxylic groups display lower electron density, which facilitates hydrogen bonding [50]. LOL further indicates weakly localized bonds between nitrogen and adjacent carbons, consistent with the low stabilization energies observed in NBO analysis. NBO analysis quantifies intramolecular conjugation effects, such as σ→π and π→π charge transfers. In the case of ETFS peptides, hydrogen bonds formed between F19 side chains and the backbone contribute to the stabilization of α-helical structures. The bond dissociation energy (H-BDE) highlights sensitivity to oxidation [12]. DFT calculations have identified five possible pathways for βAsp peptide bond cleavage, with the α-carboxyl oxygen attacking the α-amide carbon (αOc→αCN) exhibiting the lowest activation energy (29.4 kcal/mol). This pathway demonstrates significant kinetic advantages (ΔG‡ ≈ 15 kcal/mol) over others, aligning with experimental observations of γIn + 1-46 fragments. Intermediate stability is regulated by ring strain and intramolecular hydrogen bonding, while βAsp→Asp isomerization is suppressed due to high energy barriers (>40 kcal/mol) [51].

Wannier center analysis, a post-processing technique grounded in DFT, provides complementary insights into electron localization and bonding characteristics. For example, Bharath [52] examined alterations in the Cα−OH bond length and electron density during catalysis in IDH1, uncovering changes in bond polarity and stability.

In the study of large-molecule pharmaceuticals, DFT frequently serves as a complementary tool alongside other modeling techniques such as Quantum Mechanics/Molecular Mechanics/Molecular Dynamics (QM/MM/MD). For example, the B3LYP and BLYP functionals are employed within QM/MM/MD simulations to examine deprotonation and reduction processes. Additionally, Wannier centers are utilized to further analyze bond cleavage and formation during reactions, thereby facilitating research on ligand binding to kinases. In the context of COVID-19 drug modeling, DFT has played a crucial role in investigating the inhibition mechanisms of the SARS-CoV-2 main protease (Mpro) and RNA-dependent RNA polymerase (RdRp) [53]. While DFT is particularly effective for medium-sized molecular systems concerning electronic property calculations and mechanistic studies, QM/MM is favored for large biomolecular systems that require the simultaneous consideration of chemical reactions and macromolecular environments. QM-cluster methods are especially appropriate for detailed investigations of enzymatic active sites when high-precision electronic structure data is required.

In addition to its utility in elucidating drug reaction mechanisms, DFT is extensively utilized to explore the degradation mechanisms of macromolecules in vivo, providing a comprehensive assessment of these processes, including the effects of solvents. By employing the PCM and its extended variant, C-PCM, to simulate aqueous environments, DFT facilitates the calculation of activation free energy and reaction free energy, thereby enabling the evaluation of degradation pathways. Case studies demonstrate that the optimal pathway for βAsp cleavage involves αO_3_→αC_3_ cyclization, characterized by the lowest activation energy barrier, which combines both kinetic and thermodynamic advantages [51].

### 3.4. Crystal Prediction and Examination of Crystal Stability

DFT enables precise quantum chemical calculations of molecular crystal geometries, energetic characteristics, and vibrational modes. Central to the prediction of polymorphs and the assessment of their stability are thermodynamic parameters, notably the Gibbs free energy (ΔG). According to the second law of thermodynamics, the polymorph exhibiting the lowest ΔG is deemed the most thermodynamically stable. DFT evaluates the relative stability of polymorphs by calculating ΔG, which encompasses contributions from lattice energy and intermolecular interaction energies. Thermodynamic parameters, including enthalpy (ΔH), entropy (ΔS), and ΔG, are generally determined under standard conditions (298 K, 1 atm). A negative ΔG signifies an exothermic process, whereas a positive ΔG indicates a non-spontaneous reaction. The Gibbs free energy is mathematically represented as follows:

ΔG = ΔH − TΔS,(1)

where: ΔH = enthalpy change (J or kcal), T = temperature (K), ΔS = entropy change (J/K or kcal/K). DFT primarily focuses on two core research directions: crystal structure analysis and co-crystallization mechanisms.

#### 3.4.1. Crystal Structure and Polymorph Analysis

DFT validates energetically favorable crystal configurations by optimizing experimentally derived structures (e.g., from XRD or solid-state NMR) [54]. When combined with lattice energy calculations and assessments of intermolecular interactions, the stability of these configurations can be quantitatively evaluated [55]. Periodic DFT (PDFT) facilitates the generation of candidate crystal structures [56], enabling the screening of thermodynamically stable crystal forms through Gibbs energy calculations. Subtle polymorphic differences are further elucidated using Gibbs free energy and vibrational spectral analyses [57]. These methodologies provide theoretical insights into molecular packing and physicochemical properties, including solubility and mechanical behavior. Metastable polymorphs, which are characterized by higher energy due to molecular conformational flexibility, can transition to more stable forms when subjected to external energy inputs, such as grinding. This process overcomes low-energy barriers for atomic flipping or sp3-sp2 bond rotation, leading to the formation of stable polycrystalline forms with more favorable centrosymmetric supramolecular synthons [58].

#### 3.4.2. Co-Crystallization Mechanisms and Reaction Pathways

DFT provides insights into the mechanisms of co-crystal formation by examining hydrogen-bonding patterns, relative energies, and changes in electronic structure. This theoretical approach is often complemented by experimental techniques such as terahertz spectroscopy and Raman spectroscopy. For example, a comprehensive analysis of the molecular structure and hydrogen bonding in a eutectic system using DFT, terahertz time-domain spectroscopy (THz-TDS), and Raman spectroscopy demonstrated that acetamide (ETZ) and gallic acid (GA) form dimers through O−H···O hydrogen bonds [59]. The possible eutectic structure was optimized by B3LYP functional and 6-311 ++ G (d,p) basis set. The agreement between the theoretical model and the experimental results was verified by comparing with the experimental vibrational spectra, and the eutectic formed by C (N) NH... HOOC hydrogen bond between ACZ and 4HBA was determined [60]. Similarly, rifampicin (RIF) and tromethamine (TRIS) co-crystals exhibit enhanced stability due to energy-lowering hydrogen-bond networks [61].

#### 3.4.3. Co-Crystal Production Process Optimization and Reaction Prediction

In the process of co-crystal drug synthesis, enhancing the synthesis rate has consistently been a central research objective. DFT plays a pivotal role in optimizing the synthesis pathway by identifying reaction pathways with low energy barriers, thereby improving yield. Concurrently, DFT mitigates production risks by predicting charge effects and phase transition behaviors, which is instrumental in the design of process parameters and the analysis of microscopic molecular interactions.

- Yield Prediction and Process Parameter Optimization

DFT calculates critical molecular-level descriptors, including molecular orbital energy levels, atomic charge distributions, and local electron densities. These descriptors are crucial for identifying reactive sites and analyzing the stability of transition states. Subsequently, they are utilized as inputs for machine learning models, such as random forests and neural networks, which facilitate the establishment of quantitative relationships between reaction characteristics and yields. This methodology enhances the predictive capability regarding the synthesis efficiency of novel reactions [62].

Effective work function (WF) is a key parameter describing surface electronic properties, reflecting the ease of electron transfer during contact and separation. The effective WF plays a significant role in predicting triboelectric tendencies and mitigating electrostatic issues, thereby optimizing manufacturing parameters and enhancing productivity. The calculation of the work function (WF) is performed using the following equation:

WF = E_vac_ − E_F_, (2)

where E_vac_ is the vacuum energy, defined as the electrostatic potential in the vacuum gap when it reaches an asymptotic value. The Fermi energy (E_F_), the highest energy electron of the system at 0 K, is calculated at half of the energy gap.

Periodic DFT calculations have been conducted to examine the crystalline properties of aspirin and paracetamol. A generalized gradient approximation (GGA) functional, specifically the PBE (Perdew–Burke–Ernzerhof) exchange-correlation functional, was utilized. The TS (Thole’s damping) dispersion correction was incorporated to account for van der Waals interactions and hydrogen bonding effects. The BFDH (Bond-Order Consistent Force Field with Dispersion) model was applied to predict the crystalline morphologies of aspirin and paracetamol. For each crystal, the primary crystallographic faces were selected for analysis. The WF was calculated for each face. Single water molecules were adsorbed onto each surface, and the resulting changes in WF were compared between different faces and under conditions with and without water adsorption. The computational results revealed that the total WF values across all surfaces of aspirin and paracetamol crystals indicate that aspirin exhibits a higher WF, rendering it more susceptible to negative charging. Upon water adsorption, the WF increased across all surfaces, suggesting that electron removal became more challenging. This finding implies that electrons are more likely to transfer from materials with lower WF to those with higher WF. These findings highlight the importance of controlling environmental humidity to mitigate electrostatic risks during processing [63].

- Molecular Mechanism Analysis of Co-Crystallization Defects

DFT, in conjunction with thermodynamic calculations, offers significant insights into the microscopic mechanisms underlying incomplete co-crystallization and suggests strategies for process enhancement. For example, in the co-crystallization of ibuprofen and nicotinamide, DFT was utilized to compute the dimer binding energy and solvation energy of ibuprofen dimers, thereby evaluating their stability. The findings indicated that the formation of ibuprofen dimers modifies the phase diagram of the mixture, resulting in phase separation [64]. To investigate the impact of solvents (e.g., water), the COSMO model was employed. Solvation energy calculations revealed that the addition of water suppresses the formation of ibuprofen dimers, thus enhancing the homogeneity of the co-crystal. Moreover, DFT was applied to calculate the Gibbs free energy of the ibuprofen–nicotinamide mixture, and phase diagrams were subsequently constructed. The analysis of these phase diagrams demonstrated that the formation of ibuprofen dimers substantially lowers the lower critical solution temperature (LCST). At elevated temperatures, the prevalence of ibuprofen dimers increases, leading to a reduction in co-crystal yield.

Based on these findings, process optimization strategies can be implemented to improve the yield and uniformity of co-crystals. For example, selecting a temperature above the melting point of ibuprofen and incorporating water addition can effectively inhibit dimer formation, thereby improving the overall quality of the co-crystallization process.

## 4. DFT Applications in Investigating Drug Formulation Component Interactions

DFT, as a powerful computational methodology, furnishes molecular-level insights into the fundamental nature of drug–excipient interactions, thereby offering theoretical guidance for the rational design and optimization of formulations. Beyond its conventional applications in excipient screening and stability evaluation, DFT’s utility extends to a wide array of domains, including solubility enhancement, compatibility assessment, interfacial interactions, co-crystal engineering, chemical stability, targeting efficiency, and the analysis of mechanical properties.

### 4.1. Drug–Nanocarrier Interaction Mechanisms

**Figure 3:**
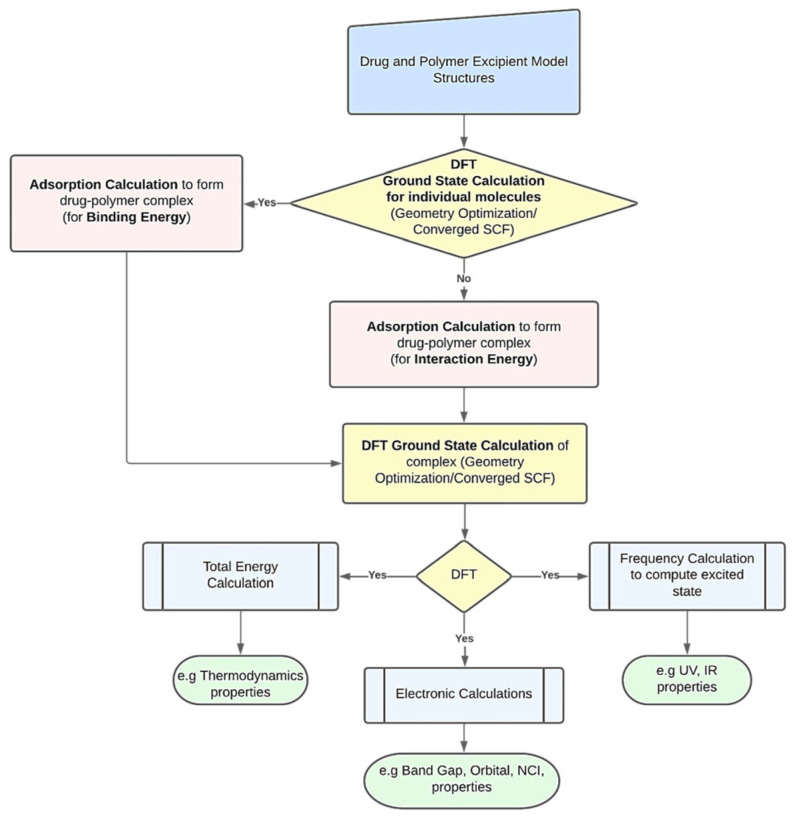
A schematic workflow for employing DFT calculations in polymer-based drug delivery systems. Reproduced from ref. [65]. This is an open-access publication.

Nanocarriers exhibit unique capabilities in targeted drug delivery and controlled release, with their intricate interaction mechanisms with drug molecules playing a pivotal role in optimizing therapeutic efficacy. DFT serves as a robust computational framework to decipher these interactions, encompassing the following key analytical dimensions:

- Adsorption energy analysis: adsorption energy (Eads), deformation energy, and interaction energy collectively quantify the binding strength between drugs and nanocarriers.
- Charge distribution profiling: molecular electrostatic potential (MEP) maps and electrostatic potential extrema identify preferential adsorption sites, guiding the rational design of nanocarriers with tailored surface properties.
- Electronic property characterization: electron transfer capacity and adsorption stability are assessed by HOMO/LUMO/energy gap, density of states analysis, and NBO.
- Thermodynamic evaluation: spontaneity and feasibility of adsorption are assessed through enthalpy change (ΔH), entropy change (ΔS), and Gibbs free energy change (ΔG). Negative ΔG values confirm thermodynamically driven adsorption processes.
- Solvent effects: the effect of solvents on the adsorption process was evaluated by solvation energy and adsorption energy in solvents.
- Drug release dynamics: the release capacity was evaluated by recovery time (τ) and protonation effects.
- In addition to the aforementioned aspects, the adsorption behavior also encompasses analyses of non-covalent interactions (NCI), Quantum Theory of Atoms in Molecules (QTAIM), and reactivity indices. The research begins with the construction and optimization of molecular structures, followed by energy minimization through structural relaxation. Subsequently, the binding energy and interaction energy of drugs on nanocarriers are calculated to predict the stability and release properties of drug–carrier complexes (see Figure 3) [65].

#### 4.1.1. Adsorption Energy Analysis

Adsorption energy is a pivotal parameter for assessing the binding strength between drug molecules and nanocarriers. Essential components include adsorption energy (E_ad_), deformation energy (E_def_), and interaction energy. A larger negative adsorption energy indicates a strong affinity of the nanocarrier for the drug. To ensure precision, the basis set superposition error (BSSE) is corrected using the counterpoise method. The Symmetry-Adapted Perturbation Theory (SAPT) further decomposes intermolecular interactions into four components: electrostatic (E_elst_), exchange (E_exch_), induction (E_ind_), and dispersion (E_disp_). The total SAPT energy (E_SAPT0_) represents the overall interaction strength, with larger absolute values indicating dominant contributions. For instance, in anti-COVID-19 drug studies, molnupiravir adsorption on B12N12 and Al12N12 nanocarriers is primarily driven by electrostatic interactions (E_elst_), with significant contributions from E_ind_ and E_disp_, while E_exch_ exhibits destabilizing effects [66]. Similarly, temozolomide adsorption on HCM-cellulose demonstrates stable non-covalent interactions (E_ad_ = −0.743 eV), accompanied by charge transfer (as revealed by NBO analysis) and altered electronic properties (e.g., HOMO–LUMO gap narrowing and UV absorption redshift) [67].

#### 4.1.2. Solvent Effects

The impact of solvents on adsorption processes is conventionally assessed by examining solvation energy and solvent-phase adsorption energy. Computational models addressing solvent effects typically utilize explicit solvent models, such as SMD, as well as polarizable continuum models, including PCM, and conductor-like screening models, such as COSMO. The SMD model explicitly integrates solvent molecules within simulations. In contrast, the PCM model approximates solvent polarization effects by employing a continuous dielectric medium, while the COSMO model, also a continuum approach, represents solvents as a continuous medium characterized by macroscopic parameters like polarizability or permittivity.

For example, in the study of temozolomide adsorption on fullerene nanocages, the SMD model was used to simulate aqueous environments, enabling accurate characterization of solute–solvent interactions. The effects of the solvent on adsorption have been investigated by calculating adsorption energy (E_Ads_) in gas and aqueous phases. The influence of the solvent on electronic structure was analyzed through shifts in HOMO and LUMO energy levels. Changes in spectral properties induced by the solvent were analyzed using electronic transition spectra derived from the time-dependent density functional theory (TD-DFT). Furthermore, Hirshfeld atomic charge analysis was utilized to investigate the effects of the solvent on charge redistribution [68]. In the study of favipiravir (FP) interactions with chitosan nanoparticles (Chit), the PCM continuum model effectively described solvent-induced electrostatic shielding. Adsorption energy (E_ad_) was calculated for FP on unmodified and modified Chit to assess solvent contributions. The FP–Chit complex exhibited a high deformation energy (E_def_ = +79.29 kcal/mol), indicating significant structural distortion post-adsorption, which negatively impacted adsorption stability. Natural Bond Orbital (NBO) analysis and Quantum Theory of Atoms in Molecules (QTAIM) confirmed hydrogen bonding as the primary interaction between FP and Chit [69].

#### 4.1.3. Drug Release Mechanism

The recovery time, represented by τ, is characterized as the theoretical period necessary for drug molecules to desorb from the surface of a nanomaterial. This parameter is a crucial indicator for assessing the efficiency of drug release. The release dynamics of drugs from carriers can be anticipated by analyzing their adsorption energies in both gaseous and aqueous phases. In particular, a lower adsorption energy signifies weaker interactions between the drug and the carrier, thereby promoting more rapid desorption. The recovery time is calculated using Equation (3).

(3) $$\tau =\frac{1}{\upsilon 0}exp(\frac{E\mathrm{ads}}{kT})$$

where *k* is the Boltzmann’s constant, and *T* and υ0 are temperature and attempt frequency.

In a study on Se-B_12_N_12_ as a delivery system for Ciclopirox (CPX), the recovery time for CPX desorption was calculated as 2.13 s using the B3PW91 functional. This rapid desorption indicates minimal hindrance to drug release. Selenium modification increased the adsorption energy from −25.60 kcal/mol (pristine B_12_N_12_) to −33.78 kcal/mol, demonstrating enhanced drug–carrier interaction without excessively restricting release [70]. In the research on the adsorption dynamics of cephalexin (CEX) on graphene ooxide/polyethylene glycol (GO/PEG) nanocomposite surfaces, the adsorption energy of CEX in the aqueous phase was lower than in the gaseous phase, suggesting weakened drug–carrier interactions in hydrated environments (e.g., wound tissue fluid). The recovery time (τ) in the aqueous phase was shorter than in the gaseous phase, confirming that hydration accelerates drug release. COSMO model simulations revealed that water molecules disrupt drug–carrier interactions, further facilitating release [71].

### 4.2. Drug–Excipient Compatibility Studies

DFT is utilized to assess drug–excipient compatibility through the examination of interaction energy, charge transfer, molecular orbital energy levels, and solvation effects. Furthermore, DFT provides insights into adsorption energy, intermolecular forces at interfaces, and the interaction mechanisms between drugs and solubilizing excipients, thereby elucidating pathways for solubility enhancement. The primary components of this research include the following:

#### 4.2.1. Host–Guest Interactions

Host–guest interactions between cyclic excipients and active pharmaceutical ingredients (APIs) offer essential insights for predicting drug delivery and controlled release mechanisms. DFT is widely employed to investigate the interactions in host–guest complexes formed by cyclodextrins (CDs) and guest molecules. The stabilization energy (E_stab_), which is defined as the energy difference between the complex and the sum of the isolated host and guest molecules, serves as a key indicator of complex stability, The stabilization energy is calculated using Equation (4):

*E*_stab_ = *E*_complex_ – (*E*_host_ + *E*_guest_) (4)

A positive *E*_stab_ indicates an unstable complex with weak interactions, whereas a negative value suggests a stable complex. Dispersion corrections (e.g., Grimme’s D3 method) are essential for accurate energy predictions. Thermodynamic parameters (enthalpy, entropy, and free energy) were further calculated to assess stability under varying conditions. Time-dependent DFT (TD-DFT) simulations revealed electronic absorption spectra of CD complexes, providing insights into guest orientation and electronic structure changes within the CD cavity. Natural bond orbital (NBO) analysis and the quantum theory of atoms in molecules (QTAIM) were applied to decode interaction mechanisms, highlighting contributions from hydrogen bonding, van der Waals forces, and dipole interactions [72].

#### 4.2.2. Chemical Activity Prediction

A comprehensive multidimensional analysis—encompassing molecular orbital energies, binding free energy, interaction forces, hydrogen bond dynamics, and structural deformation—has elucidated the differences in chemical activity between drugs and excipients. The chemical activity is predicted through DFT analysis, specifically examining the orbital energy (HOMO–LUMO energy gap, ΔE_gap) and chemical hardness (η) of frontier molecules, as well as the electrophilicity index (ω). A larger ΔE_gap typically indicates lower chemical activity, as it suggests a reduced likelihood of electronic transitions. Chemical hardness (η) represents a molecule’s resistance to polarization; thus, a higher η value implies greater difficulty in polarization and generally correlates with decreased chemical activity. The electrophilicity index (ω) quantifies a molecule’s propensity to accept electrons, thereby reflecting its electrophilic nature. The higher the ω, the easier it is for the molecule to accept electrons, which usually means stronger electron attraction and higher chemical activity. Concurrently, DFT can be integrated with MD simulations to examine and analyze the interactions between drug molecules and 2-hydroxypropyl-β-cyclodextrin (2HPβCD). This approach allows for the observation of how the drug approaches, interacts with, and eventually enters the cavity of 2HPβCD. Furthermore, the various modes of entry influence the stability and release behavior of the drug [73].

#### 4.2.3. Solubilization Mechanism Investigation

The solubilization mechanisms of excipients have been investigated using a comprehensive analysis of the HOMO–LUMO gap, solvation effects, Mulliken charges, and electrostatic potential mapping. Key parameters, including the energy gap (ΔE), dipole moment (μ), binding energy (EB), polarizability (α0), and atomic charges, were examined in mixed micellar systems such as G5^2^⁺/TX-100. The results indicated a reduction in ΔE and an increase in reactivity within the mixed systems. The greater chemical hardness of G5^2^⁺ relative to TX-100 suggested enhanced stability. Additionally, elevated electrophilicity and polarizability values were indicative of an improved electron-accepting capacity and thermodynamic stability [74]. In the investigation of the use of PEG400 and IPM as co-solvents to replace ethanol in the preparation of salbutamol sulfate (SS) suspensions based on HFA134a, χ parameters and mixing free energy (E_mix_) were calculated. The χ value is a key parameter in the Fory–Huggins model, and E_mix_ is the free energy difference between the mixed state and the pure state. A χ value approaching zero suggests that the interaction between the two molecules is near ideal, indicating enhanced solubility. Similarly, an Emix value approximating zero signifies favorable solubilization. The results demonstrated that PEG400 exhibited lower χ and Emix values compared to IPM, thereby confirming its superior compatibility [75].

#### 4.2.4. Interfacial Interactions

DFT elucidated drug–excipient interactions at solid–liquid or solid–solid interfaces by calculating adsorption energies, charge distributions, and intermolecular forces. It provides a key theoretical basis for optimizing the surface modification strategy of drug carrier materials. For example, three silica surface models—dehydroxylated (DS), fully hydroxylated (HS), and benzalkonium chloride-functionalized (FS)—were evaluated for 4-formylaminoantipyrine (FAA) adsorption Among these, the DS model demonstrated the lowest adsorption energy (−5.34 eV), with hydrogen bonding identified as the predominant interaction mechanism [76]. Additionally, DFT analysis revealed enhanced adsorption of mercaptopurine on Li/Na-decorated γ-graphyne. The charge transfer from Li to γ-graphyne modified its electronic structure, thereby facilitating stronger interactions. This was further corroborated by Density of States (DOS) analysis, which confirmed electronic redistribution, correlating with an improved adsorption capacity [77].

### 4.3. Drug–Excipient Co-Crystal Design

Binding energy calculations, derived from DFT simulations, are widely utilized to quantify interaction strengths between active pharmaceutical ingredients (APIs), co-formers, and excipients. To investigate the effects of tablet excipients on co-crystal formation and stability, the Perdew–Burke–Ernzerhof (PBE) functional combined with Grimme D3 dispersion correction was applied. Computational evaluations of five common co-formers—polyethylene glycol (PEG), hydroxypropyl methylcellulose (HPMC), polyvinylpyrrolidone (PVP), microcrystalline cellulose (MCC), and lactose—revealed that PEG exhibited the lowest binding energy, indicating its superior potential for stabilizing co-crystalline structures [78].

**Figure 4:**
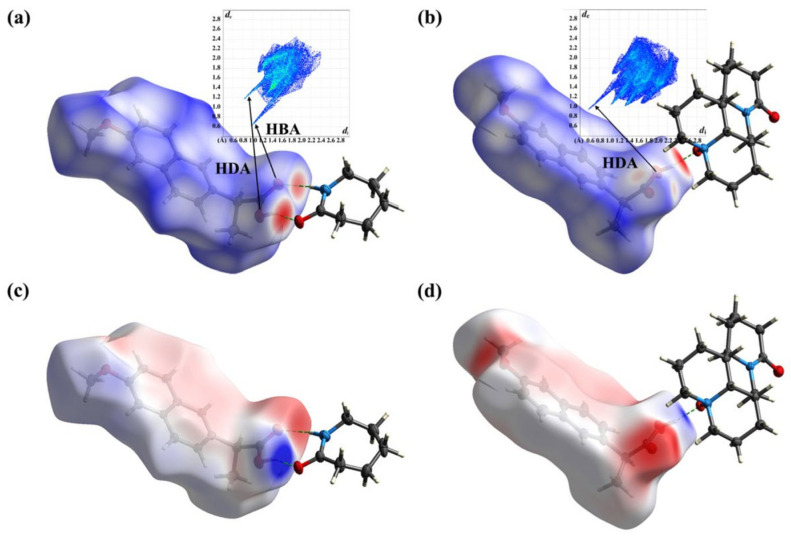
HS, FP, and MEPS analysis of NPX–CPL and NPX–OMT. (**a**) HS and FP of NPX–CPL; (**b**) HS and FP of NPX–OMT; (**c**) MEPS of NPX–CPL; (**d**) MEPS of NPX–OMT. Reproduced from ref. [80]. This is an open-access publication.

Non-covalent interactions, particularly hydrogen bonding and π-π stacking, play a crucial role in enhancing the physicochemical properties of co-crystals. These interactions are instrumental in co-former screening by influencing structural stability and functionality. As a predominant driving force, hydrogen bonds stabilize the interactions between the active pharmaceutical ingredient (API) and the co-former by reducing the system’s energy, thereby facilitating co-crystallization. They significantly enhance the thermal and chemical stability of co-crystals and modulate drug solubility. π-π stacking, although a weaker interaction, is essential as it involves the overlapping of π-electron clouds between aromatic systems. This interaction reinforces the binding between the API and the co-former, directs specific molecular packing arrangements, and works synergistically with hydrogen bonding to stabilize crystal lattices. For example, a comparative DFT analysis of two naproxen co-crystals—naproxen-oxymatrine (NPX-OMT) and naproxen-caprolactam (NPX-CPL)—employed the Independent Gradient Model based on Hirshfeld partitioning (IGMH) and molecular electrostatic potential surface (MEPS) methods. Hirshfeld surface (HS) visualization (Figure 4) highlighted hydrogen bond donor/acceptor regions and π-π interaction patterns. The dark red areas denote the formation of hydrogen bonds, with the intensity of the color correlating with bond strength. The findings revealed that NPX-OMT demonstrated enhanced solubility and thermal stability, attributed to stronger hydrogen bonds, optimized π-π stacking interactions, and favorable lattice energy in comparison to NPX-CPL [79].

### 4.4. Impact of Drug–Excipient Interactions on Drug Stability and Targeting

DFT offers detailed theoretical insights into molecular interactions, such as hydrogen bonding, van der Waals forces, and electrostatic effects, as well as alterations in electronic structure. This theoretical framework is instrumental in optimizing drug stability and informing the design of targeted therapeutics.

#### 4.4.1. Stability Implications

The chemical stability of pharmaceuticals, particularly regarding their antioxidant capacity and thermodynamic stability, is significantly affected by interactions with excipients. DFT-based analyses, which encompass hydrogen bond characterization, thermodynamic parameters (ΔE_stb_, ΔE_int_, BDE), and solvent effect simulations, provide insights into the mechanisms through which excipients enhance stability. For instance, β-Cyclodextrin (β-CD) stabilizes olive polyphenols through the formation of intermolecular O–H∙∙∙O hydrogen bonds. DFT calculations of stabilization energy (ΔE_stb_) and interaction energy (ΔE_int_), supported by DPPH radical scavenging assays, validate the enhanced antioxidant activity [80].

In the study of apigenin antioxidant activity modulated by cyclodextrin (CD) inclusion complexes, DFT-based calculations were utilized to assess three radical scavenging mechanisms: hydrogen atom transfer (HAT), single electron transfer-proton transfer (SET-PT), and sequential proton loss electron transfer (SPLET). Thermodynamic parameters, such as bond dissociation energy (BDE), ionization potential (IP), and proton affinity (PA), were utilized to characterize the impact of CD encapsulation on apigenin’s free radical scavenging efficacy. Key findings revealed solvent-dependent behavior: in polar solvents (e.g., ethanol–water mixtures), CD inclusion reduced proton affinity (PA), enhancing proton donor capacity and thereby improving antioxidant activity, while in nonpolar solvents, CD encapsulation increased BDE, IP, and PA values, suppressing antioxidant performance due to hindered proton/electron transfer processes.

Thermodynamic stabilization is a major area of research. Citalopram and escitalopram create stable inclusion complexes within β-CD dimer cavities via weak van der Waals forces. DFT calculations and single-crystal X-ray diffraction analyses confirm their crystalline symmetry, distinguishing between triclinic and monoclinic systems [81].

#### 4.4.2. Targeting Enhancement

DFT elucidates the regulatory mechanisms of excipients on drug targeting by calculating binding energies and charge distribution between targeted drugs and excipients in terms of molecular recognition and receptor binding.

DFT-based assessments of binding energy are employed to evaluate the stability and interaction strength of drug–excipient complexes during synthesis. Analyses of charge distribution elucidate the manner in which excipients alter the electronic state of drugs, subsequently affecting their interactions with biological targets. For instance, the substantial binding energy observed between mannose and punicalagin suggests the formation of a stable complex. Furthermore, hydrogen bonding and electrostatic interactions, particularly at the GLU706 and GLU719 residues, facilitate the targeting of the drug toward macrophage mannose receptors [82]. Regarding charge distribution optimization, α-Tocopheryl succinate (α-TOS) improves the target affinity of doxorubicin by modifying its electron cloud distribution through charge transfer. Combined DFT simulations and liposome models clarify excipient-driven molecular recognition mechanisms, providing insights into lipid bilayer interactions and targeted delivery pathways [83].

Selected applications of DFT discussed in the cited references are summarized in Table 1.

## 5. Application of DFT in Drug Delivery and Pharmacokinetic Studies

DFT has become an essential instrument for elucidating molecular mechanisms in drug delivery and pharmacokinetics. It provides atomic-level insights into drug–carrier interactions, release kinetics, membrane permeability, and the integration of analytical methodologies.

### 5.1. DFT in Drug Release Mechanism Studies

- Hydrogen Bonding and van der Waals Force-Mediated Sustained Release

In the study of β-cyclodextrin (β-CD)-mediated chiral recognition of D- and L-penicillamine (Pen), DFT simulations were integrated with quantum chemical topological analyses—Atoms in Molecules (AIM) and Independent Gradient Model (IGM)—to elucidate hydrogen bonding and van der Waals interactions. AIM analysis is a topological characterization that reveals intramolecular chemical bonding and critical interaction sites. The IGM analysis is an electron density gradient mapping process to identify intermolecular interaction regions. The strength of the hydrogen bond is measured by the q value and the HBCP value. L-Pen exhibited stronger hydrogen bonding with β-CD (charge transfer, q = 0.054982; HBCP = −0.009978 a.u.) compared to D-Pen (q = 0.049242; HBCP = −0.006706 a.u.), rationalizing the preferential inclusion and sustained release of L-Pen [85].

- pH-Responsive Release Mechanisms:

pH-responsive drug delivery systems leverage the physiological pH variations present across different human organs, such as the stomach (pH 1–3), intestine (pH 6–8), and tumor microenvironments (pH 5–6), to facilitate site-specific drug release. By integrating DFT and molecular dynamics (MD) simulations, researchers can quantitatively predict the interactions between drugs and carriers that are dependent on pH, as well as the kinetics of drug release. A critical aspect of studying pH-responsive drug delivery systems involves calculating the protonation states of drug molecules and carriers under different pH conditions, which is accomplished using the Henderson–Hasselbalch equation. DFT-based calculations are employed to evaluate the total interaction energies, thereby identifying the pH-triggered thresholds for adsorption and desorption. For example, in the study of the Alendronate Propylamine-Modified Silica System, it was observed that under low pH conditions (pH 1.0), a strong electrostatic attraction occurs between the protonated amine groups (-NH_3_⁺) and anionic alendronate, resulting in a maximal negative interaction energy (E_total = −70 kcal/mol), which is conducive to drug adsorption. Conversely, under high pH conditions (pH > 8.0), the deprotonation of amines (-NH_2_) alters the surface charge, leading to the generation of repulsive forces (E_total = +20 kcal/mol) and facilitating the release of the drug [84]. In the study of the polyethylenimine (PEI)-functionalized graphene-doxorubicin (DOX) system, it was observed that at a neutral pH of 7.4, synergistic O–H···N (−39.99 kJ/mol) and N–H···N (−12.59 kJ/mol) hydrogen bonds contributed to the stabilization of π-π stacking interactions between DOX and graphene, with an adsorption distance ranging from 2.51 to 3.50 Å. The total adsorption energy was calculated to be −284.08 kJ/mol, which ensures stable drug retention. Conversely, under acidic conditions (pH 5.0), the protonation of PEI amines (-NH_3_⁺) led to electrostatic repulsion and the disruption of hydrogen bonds, resulting in a decrease in adsorption energy to −160.55 kJ/mol. This reduction in energy facilitates the accelerated release of DOX in tumor microenvironments [86].

### 5.2. Application of DFT in Biomembrane Permeability and Bioavailability Prediction

DFT provides critical insights into drug–biomembrane interactions, enabling quantitative predictions of permeability and bioavailability. Key findings include hydrophobic-driven permeation and its implications for bioavailability. For instance, in the case of emerging contaminants such as triclosan, RI-BP86-D3/def2-SVP calculations demonstrated a stronger affinity for phospholipid acyl chains (ΔE = −22.2 kcal/mol) compared to polar headgroups (ΔE = −9.8 kcal/mol), thereby promoting penetration into the hydrophobic bilayer. Conversely, parabens showed a preference for binding to polar headgroups (ΔE = −31.1 kcal/mol), which impedes their deep membrane penetration [86]. These results rationalize how molecular hydrophobicity and binding site specificity govern drug–membrane partitioning, informing strategies to optimize bioavailability via structural modifications (e.g., logP tuning) [87].

### 5.3. Synergistic Integration of DFT and Solid-State NMR

The integration of DFT with experimental analytical techniques offers comprehensive theoretical support for pharmaceutical research. In nuclear magnetic resonance (NMR) analysis, DFT is primarily applied to predict chemical shifts, elucidate hydrogen bonding and isotope effects, investigate tautomerism, and analyze geometric isomers and coupling constants. The precision of these predictions is markedly improved through the application of long-range corrected functionals, such as ωB97X-D [88]. For instance, DFT calculations enable structural differentiation between hydrated and anhydrous forms of active pharmaceutical ingredients (APIs), where 35Cl solid-state NMR (SSNMR) spectroscopy serves as an ideal tool for studying crystalline APIs in dosage forms. By computing the 35Cl and 2H chemical shifts and determining the electric field gradient (EFG) tensors of chlorine atoms, DFT aids in distinguishing molecular structures under varying hydration states and analyzing hydrogen bonding interactions between water molecules and API [89]. Furthermore, combining DFT with variable-temperature (VT) 2H SSNMR experiments facilitates the investigation of dynamic behaviors of hydrates at different temperatures. In addition, DFT plays a crucial role in detecting excipient-induced interference within NMR spectra. By calculating the electric field gradient (EFG) tensors of excipients and predicting their corresponding NMR signals, DFT facilitates the effective differentiation of active pharmaceutical ingredient (API) signals from complex spectral data [90].

## 6. Conclusions and Outlook

DFT has become a pivotal tool in pharmaceutical formulation research, facilitating the detailed analysis of electronic structures to unravel complex drug–excipient interactions and establish quantitative correlations between molecular design and formulation performance. This review systematically explores the advanced applications of DFT in pharmaceutics, with a focus on elucidating molecular interactions, simulating biomolecular interfacial behaviors, and developing multiphysics-coupled models. By calculating molecular orbital energy levels, electron density distributions, and thermodynamic parameters, DFT offers novel insights into the stability, solubility, and bioavailability of drug–excipient complexes.

Despite considerable advancements, numerous challenges continue to exist in contemporary research. Firstly, achieving a balance between computational accuracy and efficiency is crucial in complex solvent environments, such as ionic liquids or high-viscosity media, where simplified models like the polarizable continuum model (PCM) may fail to adequately represent dynamic effects. Secondly, the relationship between quantum chemical descriptors, such as electron density and orbital energy levels, and macroscopic physicochemical properties, such as solubility and mechanical strength, remains insufficiently characterized. There is an absence of a universal “electronic structural fingerprint” system to effectively bridge these scales.

DFT exhibits significant potential in the development of pharmaceutical formulations. Future research should focus on integrating DFT with machine learning (ML). By training ML models using molecular descriptors derived from high-throughput DFT calculations, rapid screening of excipient libraries and optimization of synthetic pathways can be achieved, thereby advancing the digital transformation of formulation molecular engineering. Concurrently, multidisciplinary models should be developed to investigate phase transition behaviors of drug crystals under dynamic processing conditions. Enhanced solvent models (e.g., refined PCM frameworks), combined with experimental spectroscopic data, could improve predictions of drug degradation pathways and bioavailability. As computational capabilities advance and interdisciplinary approaches evolve, DFT is anticipated to play an increasingly significant role in emerging fields, including intelligent responsive delivery systems and bioelectronic drugs.

## Figures and Tables

**Table 1:** Selected articles published on the application of DFT methods. Abbreviations used in the table: Natural Bond Orbitals (NBO), Nonlinear Optics (NLO), Frontier Molecular Orbital (FMO), Electron Localization Function (ELF), Lagrangian Orbital Localization (LOL), Molecular Electrostatic Potential (MEP), Average Local Ionization Energy (ALIE), Bond Dissociation Enthalpy (BDE), Electrostatic Potential (ESP), HOMO–LUMO Gap (Egap), electronegativity (χ), chemical potential (μ), hardness (η), softness (S), charge density (q), electrophilic index (ω), softness (S), entropy (Sv), enthalpy (He), Gibbs free energy (ΔG), hydrogen bond (H-Bond), vibrational frequency (VF), adsorption energy (Ead), deformation energy (Edef), interaction energy (Eint), binding energy (Eb), polarizability (α_0_), hyperpolarizability (β_0_), Bond Critical Point (BCP).

No.	DFT Code	Functional	Basis Set	Application	Ref
1	Gaussian 09/Gaussview 5.0	B3LYP	6-311 + G(2d,p)	VF, MEP	[41]
2	Gaussian 09 W	B3LYP	6-311 ++ G(d,p)	MEP/ALIE, NBO, NLO, FMO, ELF, LOL, Egap, χ, μ, η, S, HOMO/LUMO, UV–Vis	[42]
3	Gaussian 09/Gaussview 5.0	B3LYP	6-31G(d,p), 6-311G(d,p)	MEP/ALI, H-BDE, NBO, NLO, HOMO/LUMO	[42]
4	Gaussian 09W	B3LYP	6-31 ++ G(d,p)	HOMO/LUMO, Egap, μ, η, ω, S, FMO	[43]
5	Gaussian 09	BHandHLY	6-31G(d,p) 6-31 + G(d,p)	ESP, η, ω, HOMO/LUMO	[12]
6	GAMESS	LC-BLYP	cc-pVTZ	HOMO/LUMO	[48]
7	Gaussian16W	ωB97XD	6-311 + G(d,p)	HOMO/LUMO, MEP, μ, η, S,	[49]
8	Gaussian 09	B3LYP	6-311 ++ G(d,p)	ΔG, Solvent Effect	[51]
9	Gaussian	B3LYP	def2-TZVP	MiMiC-QM/MM MD, ΔG	[52]
10	CASTEP (Academic Release Version 8.0)	PBE, PBE0	Intermediate basis set	NMR, H-bond	[54]
11	Gaussian09	B3LYP	6-31 + G(d) f	∆Estb, ∆Ecpx, ∆Eint	[55]
12	Gaussian09/Gaussian-View 5.0	ωB97XD	6-31G	MP2, CSP, Lattice Energe, Sv, He, ΔG, MEP/ALIE, H-BDE	[57]
13	Gaussian 09/Gaussview 5.0	B3LYP	6-311 ++ G (d,p)	H-bond	[59]
14	Gaussian’03	B3LYP	6-311 ++ G (d,p)	H-bond, VF	[60]
15	Gaussian 16 (G16)	B3LYP	6-311 ++ G (d,p)	vibrational modes, molecular descriptors	[62]
16	Gaussian 16	M06-2X B3LYP	6-31G	Solvent Effect, VF, Ionization Potential, Electron Affinity, Egap, η, ω, μ	[68]
17	Gaussian 16	B3LYP-D3(BJ)	6-311 + g(d,p)	Ead, Edef, Eint, μ, η, ω, DOS, HOMO/LUMO, Solvent Effect	[70]
18	DMOL3	(PBE)	6-31G	HOMO/LUMO, Eb	[74]
19	Gaussian 09W	B3LYP	6-31G	HOMO/LUMO, ΔE, μ, Eb, α_0_, β_0_, Mulliken Charges and Natural Atomic Charges	[75]
20	VASP	PBE	Plane Wave Basis Set	Ead, Bader, VF, Electron Density Distribution	[77]
21	VASP	PBE	Plane Wave Basis Set	Eb, H-bond, van der Waals	[79]
22	Gaussian 16	B3LYP-D3BJ/M06-2X	6-31G(d,p)/def2-TZVP/jun-cc-pvtz	Interaction Energy, Lattice Energy	[80]
23	Jaguar	B3LYP-D3	6-31G	VF, ESP, ALIE, NCI, Electron Density, Spin Density	[83]
24	Gaussian 09	B3LYP-D3	6-31G(d,p)	VF, q, BCP Solvent Effect	[84]

## References

[1] Amidon G.L., Lennernäs H., Shah V.P., Crison J.R. (1995). A Theoretical Basis for a Biopharmaceutic Drug Classification: The Correlation of in Vitro Drug Product Dissolution and in Vivo Bioavailability. Pharm. Res..

[2] Grimme S., Antony J., Ehrlich S., Krieg H. (2010). A Consistent and Accurate Ab Initio Parametrization of Density Functional Dispersion Correction (DFT-D) for the 94 Elements H-Pu. J. Chem. Phys..

[3] Deghady A.M., Hussein R.K., Alhamzani A.G., Mera A. (2021). Density Functional Theory and Molecular Docking Investigations of the Chemical and Antibacterial Activities for 1-(4-Hydroxyphenyl)-3-Phenylprop-2-En-1-One. Molecules.

[4] Zhang B., Asakura H., Zhang J., Zhang J., De S., Yan N. (2016). Stabilizing a Platinum1 Single-Atom Catalyst on Supported Phosphomolybdic Acid without Compromising Hydrogenation Activity. Angew. Chem. Int. Ed. Engl..

[5] Kawai S., Foster A.S., Björkman T., Nowakowska S., Björk J., Canova F.F., Gade L.H., Jung T.A., Meyer E. (2016). Van Der Waals Interactions and the Limits of Isolated Atom Models at Interfaces. Nat. Commun..

[6] Li R., Zhang X., Dong H., Li Q., Shuai Z., Hu W. (2016). Gibbs-Curie-Wulff Theorem in Organic Materials: A Case Study on the Relationship between Surface Energy and Crystal Growth. Adv. Mater..

[7] Nanev C.N. (2023). Thermodynamic and Molecular-Kinetic Considerations of the Initial Growth of Newly Born Crystals; Crystal Size Distribution; Dissolution of Small Crystals during Ostwald Ripening Due to Temperature Changes. Prog. Cryst. Growth Charact. Mater..

[8] Gould T., Hashimi Z., Kronik L., Dale S.G. (2022). Single Excitation Energies Obtained from the Ensemble “HOMO-LUMO Gap”: Exact Results and Approximations. J. Phys. Chem. Lett..

[9] Friedman R. (2022). Computational Studies of Protein–Drug Binding Affinity Changes upon Mutations in the Drug Target. WIREs Comput. Mol. Sci..

[10] Sundar S., Sandilya A.A., Priya M.H. (2021). Unraveling the Influence of Osmolytes on Water Hydrogen-Bond Network: From Local Structure to Graph Theory Analysis. J. Chem. Inf. Model..

[11] Pang R., Yu L.-J., Zhang M., Tian Z.-Q., Wu D.-Y. (2016). DFT Study of Hydrogen-Bonding Interaction, Solvation Effect, and Electric-Field Effect on Raman Spectra of Hydrated Proton. J. Phys. Chem. A.

[12] Soriano-Correa C., Vichi-Ramírez M.M., Herrera-Valencia E.E., Barrientos-Salcedo C. (2023). The Role of ETFS Amino Acids on the Stability and Inhibition of P53-MDM2 Complex of Anticancer P53-Derivatives Peptides: Density Functional Theory and Molecular Docking Studies. J. Mol. Graph. Model..

[13] Koch W., Holthausen M.C. (2001). A Chemist’s Guide to Density Functional Theory.

[14] Butera V. (2024). Density Functional Theory Methods Applied to Homogeneous and Heterogeneous Catalysis: A Short Review and a Practical User Guide. Phys. Chem. Chem. Phys..

[15] Teale A.M., Helgaker T., Savin A., Adamo C., Aradi B., Arbuznikov A.V., Ayers P.W., Baerends E.J., Barone V., Calaminici P. (2022). DFT Exchange: Sharing Perspectives on the Workhorse of Quantum Chemistry and Materials Science. Phys. Chem. Chem. Phys..

[16] Iron M.A. (2017). Evaluation of the Factors Impacting the Accuracy of 13C NMR Chemical Shift Predictions Using Density Functional Theory-The Advantage of Long-Range Corrected Functionals. J. Chem. Theory Comput..

[17] Arroyo-de Dompablo M.E., Morales-García A., Taravillo M. (2011). DFT+U Calculations of Crystal Lattice, Electronic Structure, and Phase Stability under Pressure of TiO_2_ Polymorphs. J. Chem. Phys..

[18] Wen E.C.H., Jacobse P.H., Jiang J., Wang Z., Louie S.G., Crommie M.F., Fischer F.R. (2023). Fermi-Level Engineering of Nitrogen Core-Doped Armchair Graphene Nanoribbons. J. Am. Chem. Soc..

[19] Aliabad H.A.R., Mahdavi B., Azadparvar M., Golestani R., Choopani Z. (2023). DFT Study of Sertraline Hydrochloride Antidepressant Drug. J. Mol. Model..

[20] Willow S.Y., Zeng X.C., Xantheas S.S., Kim K.S., Hirata S. (2016). Why Is MP2-Water “Cooler” and “Denser” than DFT-Water?. J. Phys. Chem. Lett..

[21] Chaloupecká E., Tyrpekl V., Bártová K., Nishiyama Y., Dračínský M. (2024). NMR Crystallography of Amino Acids. Solid. State Nucl. Magn. Reson..

[22] Moreno Yalet N., Dammig Quiña P.L., Ranea V.A. (2023). A DFT Study on the Adsorption and Dissociation of N-Nitrosodimethylamine on a Ni8 Nanocluster. J. Mol. Graph. Model..

[23] Drzewiecka-Matuszek A., Rutkowska-Zbik D. (2021). Application of TD-DFT Theory to Studying Porphyrinoid-Based Photosensitizers for Photodynamic Therapy: A Review. Molecules.

[24] Anouar E.H., Weber J.-F.F. (2013). Time-Dependent Density Functional Theory Study of UV/Vis Spectra of Natural Styrylpyrones. Spectrochim. Acta A Mol. Biomol. Spectrosc..

[25] Rezaei-Sameti M., Iraji Borojeni Z. (2023). Interaction of 5-Fluorouracil Anticancer Drug with Nucleobases: Insight from DFT, TD-DFT, and AIM Calculations. J. Biomol. Struct. Dyn..

[26] Kityk A.V. (2012). Absorption and Fluorescence Spectra of Heterocyclic Isomers from Long-Range-Corrected Density Functional Theory in Polarizable Continuum Approach. J. Phys. Chem. A.

[27] Sato T., Tsuneda T., Hirao K. (2005). A Density-Functional Study on Pi-Aromatic Interaction: Benzene Dimer and Naphthalene Dimer. J. Chem. Phys..

[28] Song J.-W., Hirosawa T., Tsuneda T., Hirao K. (2007). Long-Range Corrected Density Functional Calculations of Chemical Reactions: Redetermination of Parameter. J. Chem. Phys..

[29] Dračínský M. (2021). Analyzing Discrepancies in Chemical-Shift Predictions of Solid Pyridinium Fumarates. Molecules.

[30] Womack J.C., Mardirossian N., Head-Gordon M., Skylaris C.-K. (2016). Self-Consistent Implementation of Meta-GGA Functionals for the ONETEP Linear-Scaling Electronic Structure Package. J. Chem. Phys..

[31] Albu T.V., Swaminathan S. (2006). Hybrid Density Functional Theory with Specific Reaction Parameter: Hydrogen Abstraction Reaction of Fluoromethane by the Hydroxyl Radical. J. Phys. Chem. A.

[32] Coskun D., Jerome S.V., Friesner R.A. (2016). Evaluation of the Performance of the B3LYP, PBE0, and M06 DFT Functionals, and DBLOC-Corrected Versions, in the Calculation of Redox Potentials and Spin Splittings for Transition Metal Containing Systems. J. Chem. Theory Comput..

[33] Yan Z., Wei D., Li X., Chung L.W. (2024). Accelerating Reliable Multiscale Quantum Refinement of Protein-Drug Systems Enabled by Machine Learning. Nat. Commun..

[34] Nippa D.F., Atz K., Hohler R., Müller A.T., Marx A., Bartelmus C., Wuitschik G., Marzuoli I., Jost V., Wolfard J. (2024). Enabling Late-Stage Drug Diversification by High-Throughput Experimentation with Geometric Deep Learning. Nat. Chem..

[35] Yan W., Xu X. (2022). Analytic Gradients for the Long-Range-Corrected XYG3 Type of Doubly Hybrid Density Functionals: Theory, Implementation, and Assessment. J. Phys. Chem. A.

[36] Zhang Y., Xu X., Goddard W.A. (2009). Doubly Hybrid Density Functional for Accurate Descriptions of Nonbond Interactions, Thermochemistry, and Thermochemical Kinetics. Proc. Natl. Acad. Sci. USA.

[37] Yan W., Xu X. (2022). Accurate Prediction of Nuclear Magnetic Resonance Parameters via the XYG3 Type of Doubly Hybrid Density Functionals. J. Chem. Theory Comput..

[38] Zhang H., Liu S., You J., Liu C., Zheng S., Lu Z., Wang T., Zheng N., Shao B. (2024). Overcoming the Barrier of Orbital-Free Density Functional Theory for Molecular Systems Using Deep Learning. Nat. Comput. Sci..

[39] Dasgupta S., Lambros E., Perdew J.P., Paesani F. (2021). Elevating Density Functional Theory to Chemical Accuracy for Water Simulations through a Density-Corrected Many-Body Formalism. Nat. Commun..

[40] Erdogan T. (2021). DFT, Molecular Docking and Molecular Dynamics Simulation Studies on Some Newly Introduced Natural Products for Their Potential Use against SARS-CoV-2. J. Mol. Struct..

[41] Abraham C.S., Muthu S., Prasana J.C., Armaković S., Armaković S.J., Rizwana B.F., Geoffrey B., David R.H.A. (2019). Computational Evaluation of the Reactivity and Pharmaceutical Potential of an Organic Amine: A DFT, Molecular Dynamics Simulations and Molecular Docking Approach. Spectrochim. Acta A Mol. Biomol. Spectrosc..

[42] Mary Y.S., Mary Y.S., Bielenica A., Armaković S., Armaković S.J., Chandramohan V., Dammalli M. (2021). Investigation of the Reactivity Properties of a Thiourea Derivative with Anticancer Activity by DFT and MD Simulations. J. Mol. Model..

[43] Ayub M.A., Tyagi A.R., Srivastava S.K., Singh P. (2024). Quantum DFT Analysis and Molecular Docking Investigation of Various Potential Breast Cancer Drugs. J. Mater. Chem. B.

[44] Gheidari D., Mehrdad M., Bayat M. (2024). Synthesis, Molecular Docking Analysis, Molecular Dynamic Simulation, ADMET, DFT, and Drug Likeness Studies: Novel Indeno[1,2-b]Pyrrol-4(1H)-One as SARS-CoV-2 Main Protease Inhibitors. PLoS ONE.

[45] Babu V., Ahmed S., Rahiman A.K., Kawsar S.M.A., Berredjem M., Bhat A.R., Basha K.A. (2024). Computational Chemistry: Prediction of Compound Accessibility of Targeted Synthesized Compounds. Med. Chem..

[46] Kronik L., Stein T., Refaely-Abramson S., Baer R. (2012). Excitation Gaps of Finite-Sized Systems from Optimally Tuned Range-Separated Hybrid Functionals. J. Chem. Theory Comput..

[47] Kronik L., Kümmel S. (2018). Dielectric Screening Meets Optimally Tuned Density Functionals. Adv. Mater..

[48] Wong B.M., Hsieh T.H. (2010). Optoelectronic and Excitonic Properties of Oligoacenes: Substantial Improvements from Range-Separated Time-Dependent Density Functional Theory. J. Chem. Theory Comput..

[49] Tiwari G., Chauhan M.S., Sharma D. (2024). In Silico Study of Inhibition Activity of Boceprevir Drug against 2019-nCoV Main Protease. Z. Naturforsch. C J. Biosci..

[50] Sang-Aroon W., Phatchana R., Tontapha S., Ruangpornvisuti V. (2021). A DFT Calculation on Nonenzymatic Degradation of Isoaspartic Residue. J. Mol. Model..

[51] Raghavan B., Paulikat M., Ahmad K., Callea L., Rizzi A., Ippoliti E., Mandelli D., Bonati L., De Vivo M., Carloni P. (2023). Drug Design in the Exascale Era: A Perspective from Massively Parallel QM/MM Simulations. J. Chem. Inf. Model..

[52] Ye N., Yang Z., Liu Y. (2022). Applications of Density Functional Theory in COVID-19 Drug Modeling. Drug Discov. Today.

[53] Smalley C.J.H., Hoskyns H.E., Hughes C.E., Johnstone D.N., Willhammar T., Young M.T., Pickard C.J., Logsdail A.J., Midgley P.A., Harris K.D.M. (2022). A Structure Determination Protocol Based on Combined Analysis of 3D-ED Data, Powder XRD Data, Solid-State NMR Data and DFT-D Calculations Reveals the Structure of a New Polymorph of l-Tyrosine. Chem. Sci..

[54] Konovalova I.S., Shishkina S.V., Wyshusek M., Patzer M., Reiss G.J. (2024). Supramolecular Architecture of Theophylline Polymorphs, Monohydrate and Co-Crystals with Iodine: Study from the Energetic Viewpoint. RSC Adv..

[55] Mazurek A.H., Szeleszczuk Ł., Pisklak D.M. (2020). Periodic DFT Calculations-Review of Applications in the Pharmaceutical Sciences. Pharmaceutics.

[56] Hao X., Liu J., Ali I., Luo H., Han Y., Hu W., Liu J., He X., Li J. (2021). Ab Initio Determination of Crystal Stability of Di-p-Tolyl Disulfide. Sci. Rep..

[57] Konovalova I.S., Shaposhnyk A.M., Baumer V.N., Chalyk B.A., Shishkina S.V. (2022). Polymorphic Transition Due to Grinding: The Case of 3-[1-(Tert-Butoxycarbonyl)Azetidin-3-Yl]-1,2-Oxazole-4-Carboxylic Acid. Acta Crystallogr. B Struct. Sci. Cryst. Eng. Mater..

[58] Wan M., Fang J., Xue J., Liu J., Qin J., Hong Z., Li J., Du Y. (2022). Pharmaceutical Cocrystals of Ethenzamide: Molecular Structure Analysis Based on Vibrational Spectra and DFT Calculations. Int. J. Mol. Sci..

[59] Wang Y., Xue J., Qin J., Liu J., Du Y. (2019). Structure and Spectroscopic Characterization of Pharmaceutical Co-Crystal Formation between Acetazolamide and 4-Hydroxybenzoic Acid. Spectrochim. Acta A Mol. Biomol. Spectrosc..

[60] Queiroz L.H.S., Barros R.S., de Sousa F.F., Lage M.R., Sarraguça M.C., Ribeiro P.R.S. (2024). Preparation and Characterization of a Rifampicin Coamorphous Material with Tromethamine Coformer: An Experimental-Theoretical Study. Mol. Pharm..

[61] Raghavan P., Rago A.J., Verma P., Hassan M.M., Goshu G.M., Dombrowski A.W., Pandey A., Coley C.W., Wang Y. (2024). Incorporating Synthetic Accessibility in Drug Design: Predicting Reaction Yields of Suzuki Cross-Couplings by Leveraging AbbVie’s 15-Year Parallel Library Data Set. J. Am. Chem. Soc..

[62] Middleton J.R., Scott A.J., Storey R., Marucci M., Ghadiri M. (2023). Prediction of the Effective Work Function of Aspirin and Paracetamol Crystals by Density Functional Theory-A First-Principles Study. Cryst. Growth Des..

[63] Asgarpour Khansary M., Walker G., Shirazian S. (2020). Incomplete Cocrystalization of Ibuprofen and Nicotinamide and Its Interplay with Formation of Ibuprofen Dimer and/or Nicotinamide Dimer: A Thermodynamic Analysis Based on DFT Data. Int. J. Pharm..

[64] Asgarpour Khansary M., Shirazian S., Walker G. (2021). Molecular Engineering of Cocrystallization Process in Holt Melt Extrusion Based on Kinetics of Elementary Molecular Processes. Int. J. Pharm..

[65] Adekoya O.C., Adekoya G.J., Sadiku E.R., Hamam Y., Ray S.S. (2022). Application of DFT Calculations in Designing Polymer-Based Drug Delivery Systems: An Overview. Pharmaceutics.

[66] Ibrahim M.A.A., Rady A.-S.S.M., Mohamed L.A., Shawky A.M., Hasanin T.H.A., Sidhom P.A., Moussa N.A.M. (2023). Adsorption of Molnupiravir Anti-COVID-19 Drug over B12N12 and Al12N12 Nanocarriers: A DFT Study. J. Biomol. Struct. Dyn..

[67] Shahi M., Azarakhshi F. (2023). Theoretical Study of Interaction between Temozolomide Anticancer Drug and Hydroxyethyl Carboxymethyl Cellulose Nanocarriers for Targeted Drug Delivery by DFT Quantum Mechanical Calculation. BMC Chem..

[68] Ndjopme Wandji B.L., Tamafo Fouegue A.D., Nkungli N.K., Ntieche R.A., Wahabou A. (2022). DFT Investigation on the Application of Pure and Doped X12N12 (X = B and Al) Fullerene-like Nano-Cages toward the Adsorption of Temozolomide. R. Soc. Open Sci..

[69] Ataei S., Nemati-Kande E., Bahrami A. (2023). Quantum DFT Studies on the Drug Delivery of Favipiravir Using Pristine and Functionalized Chitosan Nanoparticles. Sci. Rep..

[70] Kaviani S., Shahab S., Sheikhi M., Potkin V., Zhou H. (2021). A DFT Study of Se-Decorated B12N12 Nanocluster as a Possible Drug Delivery System for Ciclopirox. Comput. Theor. Chem..

[71] Adekoya O.C., Adekoya G.J., Sadiku R.E., Hamam Y., Ray S.S. (2022). Density Functional Theory Interaction Study of a Polyethylene Glycol-Based Nanocomposite with Cephalexin Drug for the Elimination of Wound Infection. ACS Omega.

[72] Mazurek A.H., Szeleszczuk Ł. (2022). Current Status of Quantum Chemical Studies of Cyclodextrin Host-Guest Complexes. Molecules.

[73] Ganjali Koli M., Eshaghi Malekshah R., Hajiabadi H. (2023). Insights from Molecular Dynamics and DFT Calculations into the Interaction of 1,4-Benzodiazepines with 2-Hydroxypropyl-βCD in a Theoretical Study. Sci. Rep..

[74] Azum N., Rub M.A., Chani M.T.S., Alzahrani K.A., Asad M., Kamal T. (2025). Mixed Micellization and Density Functional Theory (DFT) Studies on the Molecular Interactions between Gemini and Nonionic Surfactants. J. Oleo Sci..

[75] Aldabet A., Miller J.F., Soltani S., Golgoun S., Haroun M., Alkhayer M., Abdelwahed W. (2022). Development of an Ethanol-Free Salbutamol Sulfate Metered-Dose Inhaler: Application of Molecular Dynamic Simulation-Based Prediction of Intermolecular Interaction. Eur. J. Pharm. Biopharm..

[76] Simonetti S., Compañy A.D., Brizuela G., Juan A. (2016). β-Cristobalite (001) Surface as 4-Formaminoantipyrine Adsorbent: First Principle Study of the Effect on Adsorption of Surface Modification. Colloids Surf. B Biointerfaces.

[77] Pallikkara Chandrasekharan S., Lakshmy S., Sanyal G., Kalarikkal N., Trivedi R., Chakraborty B. (2023). Metal-Decorated γ-Graphyne as a Drug Transporting Agent for the Mercaptopurine Chemotherapy Drug: A DFT Study. Phys. Chem. Chem. Phys..

[78] Shaikh R., Shirazian S., Guerin S., Sheehan E., Thompson D., Walker G.M., Croker D.M. (2021). Understanding Solid-State Processing of Pharmaceutical Cocrystals via Milling: Role of Tablet Excipients. Int. J. Pharm..

[79] Xing C., Chen T., Wang L., An Q., Jin Y., Yang D., Zhang L., Du G., Lu Y. (2022). Two Novel Co-Crystals of Naproxen: Comparison of Stability, Solubility and Intermolecular Interaction. Pharmaceuticals.

[80] Aree T., Jongrungruangchok S. (2018). Structure-Antioxidant Activity Relationship of β-Cyclodextrin Inclusion Complexes with Olive Tyrosol, Hydroxytyrosol and Oleuropein: Deep Insights from X-Ray Analysis, DFT Calculation and DPPH Assay. Carbohydr. Polym..

[81] Aree T. (2024). Supramolecular Assemblies of Citalopram and Escitalopram in β-Cyclodextrin Dimeric Cavity: Crystallographic and Theoretical Insights. Carbohydr. Polym..

[82] Karwasra R., Ahmad S., Bano N., Qazi S., Raza K., Singh S., Varma S. (2022). Macrophage-Targeted Punicalagin Nanoengineering to Alleviate Methotrexate-Induced Neutropenia: A Molecular Docking, DFT, and MD Simulation Analysis. Molecules.

[83] Song M., Wang J., Lei J., Peng G., Zhang W., Zhang Y., Yin M., Li J., Liu Y., Wei X. (2019). Preparation and Evaluation of Liposomes Co-Loaded with Doxorubicin, Phospholipase D Inhibitor 5-Fluoro-2-Indolyl Deschlorohalopemide (FIPI) and D-Alpha Tocopheryl Acid Succinate (α-TOS) for Anti-Metastasis. Nanoscale Res. Lett..

[84] Ahmed K., Inamdar S.N., Rohman N., Skelton A.A. (2021). Acidity Constant and DFT-Based Modelling of pH-Responsive Alendronate Loading and Releasing on Propylamine-Modified Silica Surface. Phys. Chem. Chem. Phys..

[85] Huang F., Zhuang S., Liu W., Lin L., Sun L. (2021). Computational Investigation on the Chiral Differentiation of D- and L-Penicillamine by β-Cyclodextrin. Spectrochim. Acta A Mol. Biomol. Spectrosc..

[86] Alinejad A., Raissi H., Hashemzadeh H. (2020). Development and Evaluation of a pH-Responsive and Water-Soluble Drug Delivery System Based on Smart Polymer Coating of Graphene Nanosheets: An in Silico Study. RSC Adv..

[87] Oliver M., Bauzá A., Frontera A., Miró M. (2016). Fluorescent Lipid Nanoparticles as Biomembrane Models for Exploring Emerging Contaminant Bioavailability Supported by Density Functional Theory Calculations. Environ. Sci. Technol..

[88] Hansen P.E. (2024). The Synergy between Nuclear Magnetic Resonance and Density Functional Theory Calculations. Molecules.

[89] Holmes S.T., Vojvodin C.S., Veinberg N., Iacobelli E.M., Hirsh D.A., Schurko R.W. (2022). Hydrates of Active Pharmaceutical Ingredients: A 35Cl and 2H Solid-State NMR and DFT Study. Solid. State Nucl. Magn. Reson..

[90] Guan H.-Y., Feng Y.-F., Sun B.-H., Niu J.-Z., Zhang Q.-S. (2022). NMR Assignments of Six Asymmetrical N-Nitrosamine Isomers Determined in an Active Pharmaceutical Ingredient by DFT Calculations. Molecules.

